# The effect of a pre-procedure information video on anxiety levels in patients undergoing hysterosalpingography: A prospective case-control study

**DOI:** 10.4274/jtgga.2017.0118

**Published:** 2018-08-06

**Authors:** Selçuk Erkılınç, Nazlı Aksoy Kala, Meryem Kuru Pekcan, Ali İrfan Güzel, Mehmet Çınar, Nafiye Yılmaz

**Affiliations:** 1 Clinic of Gynecologic Oncology, University of Health Sciences, Tepecik Training and Research Hospital, İzmir, Turkey; 2 Clinic of Gynecology and Obstetrics, University of Health Sciences, Ankara Numune Training and Research Hospital, Ankara, Tukey; 3 Clinic of Gynecology and Obstetrics, University of Health Sciences, Zekai Tahir Burak Women’s Health Training and Research Hospital, Ankara, Turkey

**Keywords:** Hysterosalpingography, anxiety, beck anxiety inventory

## Abstract

**Objective:**

To evaluate the effect of a pre-procedural information video on anxiety levels in patients undergoing hysterosalpingography (HSG).

**Material and Methods:**

Among a total of 131 primary or secondary infertile patients, 66 were shown an information video and 67 control patients received standard care between August 2014 and January 2016. The video included information on the procedure, personnel, and the room for the procedure; the video was shown on the morning of the procedure. Patients were randomized using the complete randomization technique through which patients were included in the study and control groups week by week, randomly. The Beck Anxiety Inventory scale was conducted to the patients one hour before the procedure

**Results:**

There were no differences in demographic data. The history of previous gynecologic operations was higher in the control group. The Beck Anxiety score was significantly lower in the study group compared with the control group (6 vs 10).

**Conclusion:**

Our findings suggest that as an easy intervention to implement, a pre-procedural video education may be a beneficial tool for the management of HSG-related anxiety.

## Introduction

Infertility is one of the most prevalent diseases that affects young adults, defined as one year of attempted conception without success (1). Hysterosalpingography (HSG) is an ancillary diagnostic method for evaluating the uterine cavity and tubes (2). Infertility and congenital Mullerian anomalies are the main indications for HSG. Although there are alternative techniques such as hydrosonography, HSG is now widely used in the evaluation of infertility (2). HSG is recommended for the evaluation of the fallopian tubes as the gold standard (3). 

Although HSG is one of the most beneficial ancillary tests in the evaluation of infertility, the pain reported by patients is a critical disadvantage of the method. Up to 72% of patients reported pain during the procedure (4). The causes of the pain were reported to be peritoneal irritation caused by contrast media, uterine dilatation, and downward traction of the uterine cervix (5). The perception of pain, however, is barely affected by anatomic and physical factors. Cicinelli (6) reported social and psychological status such as depression and anxiety was a factor in the perception of pain during gynecologic invasive procedures. Women undergoing HSG evaluations have also been reported to have considerable stress and anxiety in correlation with invasiveness of the procedure (7). Furthermore, infertility in itself is an independent stressor and a source of anxiety and depression and levels of anxiety correlate with the duration of infertility (8). Video education was found to reduce pre-procedural anxiety levels in patients undergoing cardiac catheterization (9). The video included information on the cardiac catheterization procedure. The authors commented that a pre-procedural information video could be used for reducing peri-procedural anxiety. 

In light of information in the literature, our hypothesis was that a pre-procedural video would have a positive effect on anxiety levels in patients undergoing HSG. Therefore, in the current study, we aimed to evaluate the effect of an educational video on anxiety levels of patients undergoing HSG for infertility.

## Material and Methods

### Design

This study was conducted at the Department of Infertility and Reproductive Medicine between August 2014 and January 2016 after approval was received from the institutional review board. Patients were asked to participate in the study voluntarily. All patients consented to participate in the study and signed informed consent forms. The principles written in Declaration of Helsinki were followed. The patients had primary or secondary infertility. Infertility was regarded as attempting to have children for one year without conception. Patients undergoing HSG for the investigation of uterine malformations regardless of infertility treatment were excluded. Patients with a psychiatric disease, treatment with anxiolytic medication, and hearing problems in the video group were also excluded.

### Measures

The Beck Anxiety Inventory (BAI) (10) was the objective measure for anxiety levels. This inventory is a self-report questionnaire containing 21 questions related with how much each symptom bothered the patients. The patients answered the questions on the inventory scale, which were graded between *not at all* (0) and *severe* (3). The total score ranged from 0 to 63. Similar cut-off scores with a previous study on gastrointestinal endoscopy was used for the classification of anxiety (11). The classification of anxiety scores was as follows; 0-7, minimal; 8-15, mild; 16-25, moderate; and 26-63, severe anxiety. The data collected were: age, type of infertility, gravidity, parity, history of gynecologic operation, family type, income, and educational status. The BAI (10) was applied to all patients one hour before the HSG procedure.

### Procedure

The patients were scheduled for HSG after their first appointment at the infertility outpatient clinic. After obtaining informed consent, the information video was shown on a computer to the patients who were selected for the study group. On the morning of the procedure, the video was shown to all patients together who were scheduled on the same day. The surgeon was present in the room for possible questions from the patients. Patients who were selected for the control group received standard care; they received verbal information about the type, risks and benefits of the procedure by the surgeon.

### Video content

A video was recorded using a hand-held camera by one of the authors. The presenter was a female medical doctor who dressed in a white coat. The HSG procedure and risk factors, the waiting room, staff, room for the procedure, table and instruments for the procedure, and when and how to take the results were introduced to the patients.

### Patient selection

During the 6 months of the study, patients were included in the study or control groups week by week. A total of 227 patients underwent HSG during the study period and all patients were invited to participate in the study; 55 refused. The number of patients enrolled in the study was 172. Patients with psychiatric disorders, anxiolytic medication use, illiteracy, and hearing-impairment were excluded. One hundred thirty-three patients were eligible for the study. Patients scheduled for first week were proposed to participate in the video group and the those in the second week were included in the control group. At the fourth month of the study, the control group contained 66 patients, in the following two months, all patients were offered participation in the study group.

### Statistical analysis

The Kolmogorov-Smirnov or Shapiro-Wilk tests were used to test the normality of continuous data. The comparison of the continuous data was performed using the independent-sample t-test or Mann-Whitney U test where suitable. The chi-square test was performed for analyzing categorical data. P values <0.05 were considered as significant. Statistical analysis was performed using the SPSS statistics for Windows version 21.0. software package (Armonk, NY: IBM Corp.). Power analysis was performed using the G*power software package (Faul and Erdfelder 1998 Universitat Kiel, Germany).

## Results

The mean age of the study and control groups were 26.1 years and 26.8 years, respectively; there was no difference between the groups. The study and control groups showed similar results in terms of family type; living in nuclear families was the most prevalent type in both groups. The rate of patients who graduated from university in the study and control groups was 16% and 18%, respectively; similar educational status was observed in both groups. Another socioeconomic indicator, family income, was similar in the stratified income groups. Therefore, all demographic and social data investigated between the study and control groups were similar. There were no differences in terms of primary or secondary infertility. History of gynecologic surgery including myomectomy, ovarian cystectomy, diagnostic laparoscopy was present in 6% of the study group and 22% of the control group; the number of previous gynecologic procedures was significantly higher in the control group (p<0.05). Patients in the study group had significantly lower BAI scores than those in the control group (6 vs 20, respectively). The comparison of the study and control group is shown on Table 1. A Post hoc power analysis was performed. The sample sizes in the study and control groups were used for statistical power analysis. The post-hoc statistical power analysis revealed an adequate power 0.88 at large size effect. 

**Figure 1:**
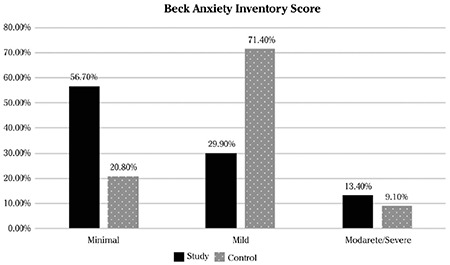
Comparison of Beck Anxiety Inventory

The severity of anxiety scores was stratified as minimal, moderate, and severe, and compared between the study and control groups. The minimal anxiety score was significantly higher in study group than in the controls (56.7% vs 20.8%). Inversely, mild anxiety was higher in the control group compared with study group (71.4 vs 29.9). No significant difference was found regarding severe anxiety. The comparison of the groups in terms of the severity of anxiety is presented in Table 2 and Figure 1.

## Discussion

This prospective case control study was designed to assess the effectiveness of an information video on reducing anxiety levels in patients undergoing HSG. The main finding of the study was that lower anxiety scores were observed in patients who watched the information video about the procedure.

HSG was reported to be related with fear and anxiety in patients (12). Various tools for reducing procedure-related anxiety such as music and medication have been identified (13,14). Group education was another intervention for reducing HSG-related anxiety. La Fianza et al. (12) investigated the importance of group education on reducing anxiety levels in women who underwent HSG, and in their preliminary report, they found a positive effect of group education on anxiety levels. Similarly, in the current study, the patients watched the educational video as a group. The patients in our study may have further benefited from group education in addition to the positive effects of video education.

Patient education with a video before a procedure was investigated in previous studies (15,16). Goodman et al. (15) found that video education had a positive effect on parents whose children had influenza vaccination. The use of an information video was investigated by Ruffinengo et al. (16) who highly recommended it as an instrument to reduce anxiety levels in the cardiology department. Similarly, our findings suggest that lower anxiety levels resulted after using a pre-procedural informative video. It is valuable that it supplies lower levels of anxiety without using medication. There is evidence that anxiolytics are useful in reducing anxiety levels in patients undergoing HSG. Women prescribed valeric acid as an anxiolytic were reported to have reduced anxiety levels (13). However, the use of these drugs is limited because of adverse effects. Another method for reducing anxiety is listening to music during the procedure. Women who listened to music during HSG were reported to have lesser anxiety levels (14). Both these studies used different anxiety score scales so it is not possible to make a comment as to which was best; prospective studies for the comparison of anxiolytics, music and information videos may reveal valuable information regarding which method is the most effective.

What should an educational video include for reducing anxiety? Chair et al. (17) investigated the effect of an information video in patients undergoing cardiac catheterization. The video included information on cardiac catheterization, the room, and staff for the procedure. Similar to our study, they found low levels of anxiety in the study population compared with the controls. Freeman-Wang et al. (18) used an information video to reduce anxiety in patents attending a colposcopy clinic. Their video included images from the reception area, nursing and medical staff in order to familiarize the patients with the department (18). Uncertainty and unfamiliarity were found to cause anxiety in patients who underwent invasive procedures (19).

In this study, the video content had certain information about HSG. The low levels of anxiety observed in the current study may be related with study group patients’ familiarity with the procedure and place where they underwent the procedure. Patient education may be performed through various routes. Patient education was reported to reduce the level anxiety regardless of the route of intervention (17). Video education was found to have more favorable psychological effects than standard education with brochures (19). In the current study, standard care was communication with the patient about the types, risks and benefits of the procedure, and our findings were compatible with the literature.

Although severe anxiety did not differ between the groups, mild anxiety was less common in the study group. Intervention with an information video might not be sufficient for effecting severe anxiety, however. Further strategies may be needed for managing sever anxiety in patients undergoing HSG. Nevertheless, the information video may a sufficient for managing mild level of anxiety.

The strong points of this study are the use of standardized tools (BAI) and adequate statistical power with large effect size.

The study was limited by the lack of true randomization. A future study recruiting randomized patients to the groups can be conducted. The higher numbers of previous gynecologic operations in the study group was a confounding factor and might have interfered with the higher levels of anxiety in the study group.

In conclusion, our findings suggest that implementing a pre-procedural education video is an easy intervention that may be used as a beneficial tool for reducing HSG-related anxiety.

## Figures and Tables

**Table 1:**
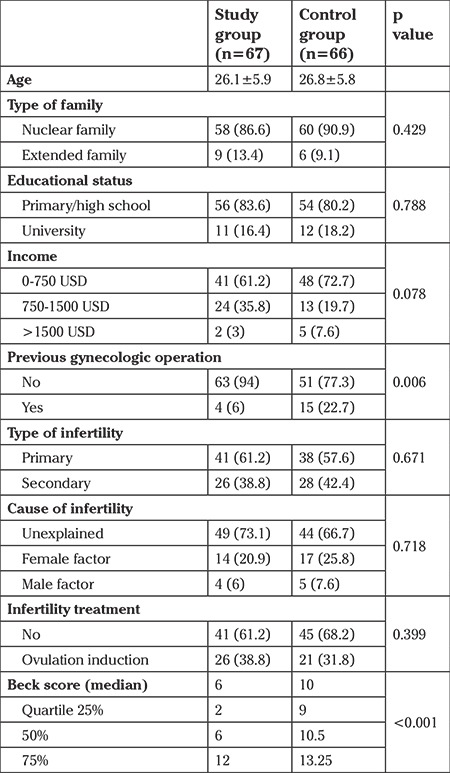
Comparison of the data between study and control groups

**Table 2:**
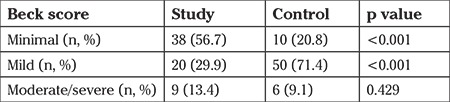
Beck Anxiety Inventory in study and control groups
